# Distinct calcium responses distinguish itch- and pain-associated neurons in the anterior cingulate cortex

**DOI:** 10.3389/fnbeh.2026.1948042

**Published:** 2026-10-27

**Authors:** Chiwoo Lee, Hyoung-Gon Ko

**Affiliations:** Department of Oral Anatomy and Neurobiology, Kyung Hee University College of Dentistry, Seoul, Republic of Korea

**Keywords:** anterior cingulate cortex, itch, pain, calcium imaging, calcium transient

## Abstract

Itch and pain are distinct aversive sensations. We previously identified segregated neuronal populations in the anterior cingulate cortex (ACC) that selectively respond to pain and itch stimuli and reported full-trace classification with a mean-frequency difference, leaving the broader calcium-transient properties associated with this separation unresolved. Here, we reanalyzed denoised *in vivo* calcium traces from stimulus-responsive ACC neurons and quantified interpretable response features, including peak rate, peak interval, rise and decay area under the curve (AUC), and rise and decay slope. Pain- and itch-associated neurons differed across multiple single-neuron calcium features. Trace-based stochastic gradient descent (SGD) classification separated the two groups, indicating that their response profiles contain label-discriminative information. Notably, classification performance was only weakly affected by neuron-wise *z*-scoring, suggesting that separation is not explained solely by simple amplitude scaling. A mouse-constrained shuffled-label control reduced classification performance, supporting the contribution of within-mouse label-associated structure while also highlighting mouse-level sampling constraints. Paired mouse summaries and mouse-exclusive validation showed limited cross-animal consistency, whereas within-mouse neighborhood analysis retained local label-associated organization. UMAP of full traces provided descriptive visualization of neuron-level response organization. Together, these results show that interpretable calcium response features carry meaningful itch−/pain-associated structure among the analyzed responsive ACC neurons.

## 1 Introduction

Itch and pain are distinct aversive sensory states, yet they recruit partially overlapping peripheral, spinal, and supraspinal pathways. The anterior cingulate cortex (ACC) is implicated in aversive sensory and affective processing, and recent work has suggested that itch and pain can be represented by modality-specific neuronal populations in the ACC (Fuchs et al., 2014; Ko et al., 2025). The neurons activated by pain and itch stimuli overlap to a limited extent, and each stimulus-specific population receives segregated presynaptic input from distinct mediodorsal (MD) neurons. Pain-associated and itch-associated ACC populations also showed stimulus-selective functional responses in the source study (Ko et al., 2025). That study reported a mean firing-frequency difference and machine-learning classification of complete neuron-level traces, establishing condition-associated response-profile differences. The remaining analytical question concerns which interpretable single-neuron calcium-transient features accompany that separation.

Calcium imaging data contain several separable levels of information. A condition-associated difference may reflect overall fluorescence scale, event frequency, peak timing, rise-to-peak kinetics, decay-related dynamics, or combinations of these properties. A classifier trained on the full trace can use any mixture of these dimensions. The key issue, therefore, is not just whether classification succeeds, but whether the separation remains linked to interpretable signal features when simpler explanations such as global amplitude scale or mouse identity are constrained. A second constraint is the nested structure of the dataset. Multiple neurons are sampled from the same mouse, and neuron-level observations are therefore not equivalent to independent animals. This issue is a known source of pseudoreplication in neuroscience and physiology when cell-level observations are interpreted as animal-level evidence without appropriate caution (Lazic, 2010; Eisner, 2021). This study therefore treats the primary unit as the neuron-level response profile and uses mouse-aware controls as boundary-condition tests rather than as the main result.

Here, we reanalyzed calcium imaging data from post-injection responsive ACC neurons assigned to itch- or pain-evoking conditions. We first quantified interpretable calcium-response features, including group-mean response trajectory, number of detected peaks per second, peak interval, rise AUC, decay AUC, rise slope, and decay slope. We then tested whether complete neuron-level traces were sufficient for itch/pain-associated classification using SGD. Finally, we used neuron-wise *z*-scoring, mouse-constrained label shuffling, and UMAP of full traces to evaluate amplitude-scale and mouse-related explanations. These analyses characterize neuron-level calcium-response profiles; whole-animal transfer, spike-level dynamics, and causal mechanisms require separate evidence. Ko et al. (2025) established full-trace classification, a mean-frequency difference, and circuit-level segregation, whereas the present study resolves additional event-timing and rise/decay features and evaluates them under mouse-level aggregation, mouse-exclusive validation, and within-mouse controls.

## 2 Methods

### 2.1 Dataset, calcium imaging acquisition, and preprocessing

ACC calcium imaging data were reanalyzed from the denoised neuron-level calcium-trace dataset generated in the previously published ACC itch/pain study (Ko et al., 2025). The present study is a secondary analysis of the post-injection responsive neurons from that dataset. Histamine injection was used as the itch-evoking condition, and formalin injection was used as the pain-evoking condition (Hunskaar and Hole, 1987; Bell et al., 2004). In the original imaging workflow, one-photon miniscope calcium movies were captured in freely moving mice using the Miniscope DAQ software and hardware described in Ko et al., 2025, saved as AVI files, and processed with MIN1PIPE for miniscope one-photon calcium-imaging signal extraction (Lu et al., 2018). Neural populations detected across sessions were tracked with CellReg (Sheintuch et al., 2017). The present analysis began from the resulting denoised and registered neuron-level trace matrices and used the analysis steps described below for feature extraction, z-scoring, shuffled-label controls, UMAP visualization, figure generation, and Weka SGD reproduction. Experimental procedures, including mouse strain, sex and age, calcium indicator, imaging modality, ACC subregion and layer, injection site and dose, recording duration, and response-selection criteria, were as described in Ko et al., 2025.

The source experiment expressed AAV2/1-EF1alpha-GCaMP6f in the right ACC and acquired one-photon miniscope recordings from freely moving mice at 15 frames/s, corresponding to an inter-frame interval of 66.7 ms. The experimental sequence comprised 5 min of baseline imaging, brief isoflurane anesthesia and histamine or formalin injection, 5 min of recovery, and 10 min of post-injection imaging. The analyzed matrices contain 8,438 post-injection samples per neuron, corresponding to 562.533 s by sample-count exposure; time zero marks the start of the post-injection recording rather than injection. Histamine elicited scratching, whereas formalin elicited licking, shaking, and lifting in the source experiment, supporting their use as itch-evoking and pain-evoking experimental conditions (Hunskaar and Hole, 1987; Ko et al., 2025).

### 2.2 Neuron inclusion and analysis unit

The analysis was restricted to post-injection responsive ACC neurons. Neurons active before injection were excluded to focus the analysis on evoked response profiles. Each analyzed neuron inherited the condition label of its recording, either itch-associated or pain-associated. The final neuron-level dataset contained 3,160 responsive neurons from 19 mice, including 2,005 itch-associated neurons and 1,155 pain-associated neurons. The primary analysis unit was the neuron-level response profile. Mouse-level structure was evaluated as a control and interpretive boundary condition. The source cohort comprised 20 mice, whereas the supplied trace matrices contained 19 mice. Source M16 was absent from these matrices before the present reanalysis, and no additional mouse was excluded in the present study. Fourteen mice contained neurons from both labels and five mice contained Itch-labeled neurons only. Rows represented neurons nested within mice.

### 2.3 Calcium trace preprocessing and normalization

The present analysis used the denoised neuron-by-time trace matrices produced by the original calcium-imaging pipeline, after movie-level preprocessing, cell extraction, and cross-session registration in the original dataset. Raw denoised traces were used for the main feature analyses and trace-based classification. For *z*-scored analyses, each neuron’s trace was normalized across time by subtracting that neuron’s temporal mean and dividing by that neuron’s temporal standard deviation. This transformation removes neuron-specific mean and variance scale while preserving normalized temporal response structure. Peak-normalized transient shape served as a shape-normalized visualization because baseline-centering followed by peak normalization is invariant to neuron-wise *z*-scoring.

### 2.4 Peak detection and feature extraction

Peaks were detected from a 5-frame moving-average trace (333.3 ms) using a neuron-wise mean + 2 SD threshold. A detected event was required to remain above threshold for at least 3 consecutive frames (200.0 ms), and events separated by 5 frames or fewer (333.3 ms) were merged. Within each suprathreshold event segment, the peak was defined as the local maximum of the smoothed trace. Peak-to-peak interval was calculated from successive detected peak indices. For each event, rise features were computed from the local minimum within 30 frames (2.0 s) before the peak to the peak, and decay features were computed from the peak to the local minimum within 30 frames (2.0 s) after the peak, bounded by the next event onset when applicable. Rise AUC and decay AUC were calculated by trapezoidal integration after subtracting the corresponding onset or offset baseline, and rise and decay slopes were calculated as the fluorescence change divided by elapsed time. Neuron-level rise AUC, decay AUC, rise slope, decay slope, and peak interval were summarized as the median across events for each neuron. These features were chosen because they map onto interpretable dimensions of the calcium response: event frequency, event timing, rise-to-peak accumulation, post-peak decay accumulation, rise steepness, and decay steepness. Detected events are termed calcium peaks and represent fluorescence transients rather than directly observed action potentials. Representative quality-control neurons were required to contain at least two detected events and were ranked within each label by absolute deviation from the label-specific median peak rate, with at most one neuron retained per mouse and ties ordered by neuron ID. Figure 1B uses the first-ranked neuron from each label and the 60-s window containing the greatest number of accepted peaks; Supplementary Figure S1 uses the first three ranked neurons per label and displays the first 180 s. Both displays include denoised inputs, smoothed traces, neuron-specific thresholds, and accepted peaks.

**Figure 1:**
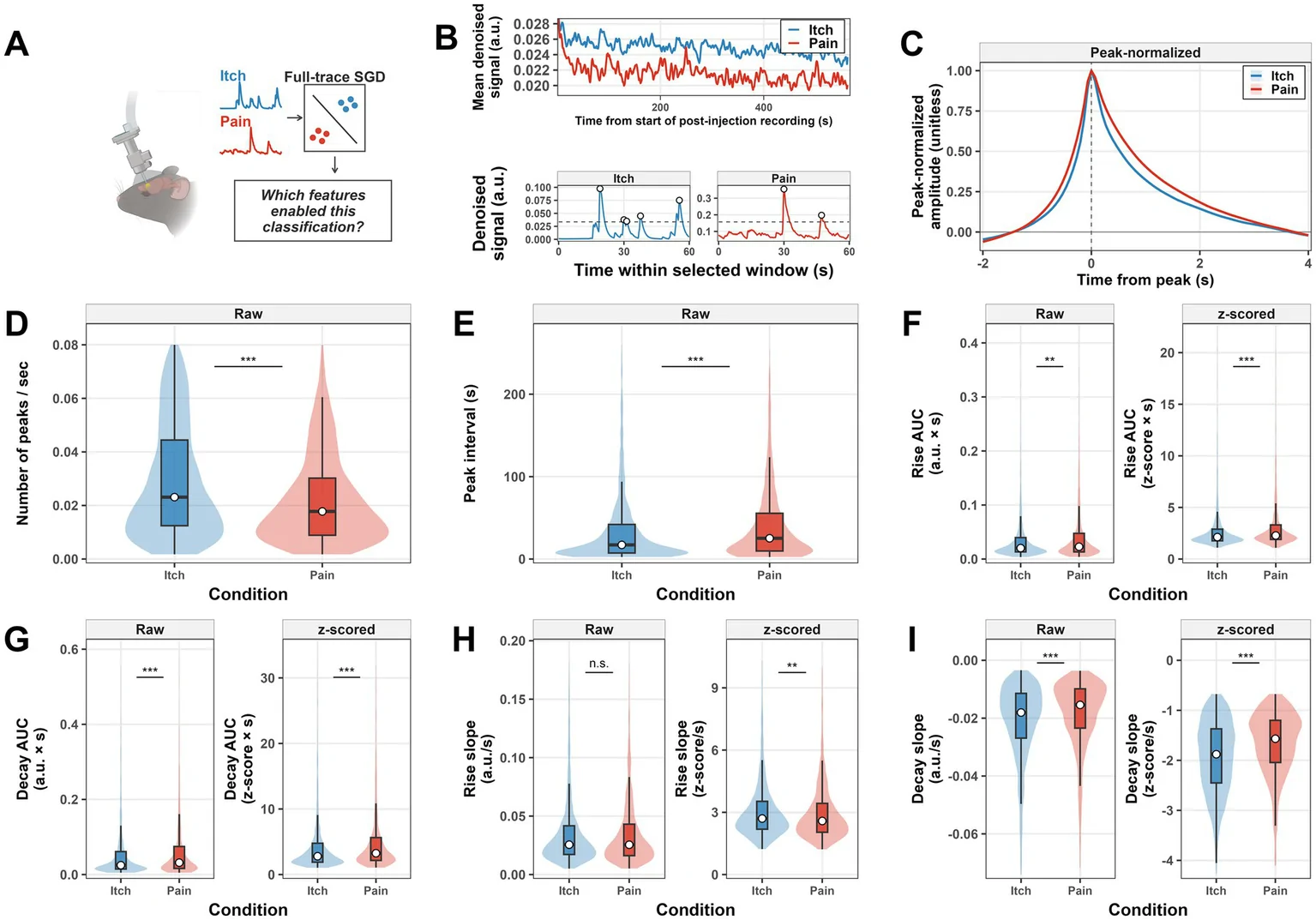
Interpretable neuron-level ACC calcium-response features accompanying the previously reported full-trace separation. **(A)** Analytical rationale. The previously reported full-trace SGD separation motivated feature-level analysis of individual-neuron calcium responses. The source data were acquired by one-photon miniscope imaging in freely moving mice; the mouse-miniscope illustration in panel **(A)** was created with BioRender.com. **(B)** The upper plot shows the group-average raw denoised signal over the analyzed post-injection interval; time zero marks the first post-injection sample rather than injection. Event timing varies across neurons, so averaging attenuates individual calcium peaks. The lower plots show 60-s windows from one itch-labeled and one pain-labeled neuron. These neurons and windows were selected by the deterministic QC rule described in Methods. Faint gray lines indicate denoised inputs, colored lines indicate five-frame smoothed signals, dashed lines indicate neuron-specific mean-plus-two-standard-deviation thresholds, and open circles mark accepted calcium peaks. **(C)** Peak-aligned calcium transients after baseline centering and normalization to the mean transient amplitude at the aligned peak. **(D–E)** Raw neuron-level distributions of peak rate and peak-to-peak interval. The corresponding *z*-scored values were identical because neuron-wise *z*-scoring preserved the accepted peak indices. **(F–I)** Neuron-level distributions of rise AUC, decay AUC, rise slope, and decay slope, each shown for raw denoised and neuron-wise *z*-scored traces. Blue indicates itch and red indicates pain. Statistical annotations show BH-adjusted two-sided Wilcoxon rank-sum tests for each displayed representation (^*^p < 0.05, ^**^p < 0.01, ^***^p < 0.001; n.s., not significant). Detected events are calcium peaks rather than directly observed action potentials.

### 2.5 Feature statistics

Neuron-level itch versus pain feature comparisons were analyzed with two-sided Wilcoxon rank-sum tests. *p* values were corrected across the six prespecified feature comparisons using the Benjamini–Hochberg (BH) procedure (Benjamini and Hochberg, 1995). Statistical annotations indicate BH-adjusted *p* values (^*^p < 0.05, ^**^p < 0.01, ^***^p < 0.001; n.s., not significant). Rank-biserial correlation was also calculated for each comparison as a nonparametric effect-size estimate, with positive values indicating larger values in itch-associated neurons and negative values indicating larger values in pain-associated neurons. These tests and effect sizes provide descriptive neuron-level comparisons rather than mouse-level inferential tests.

For mouse-level comparison, each feature was summarized by the median across neurons within each mouse-label combination. The 14 mice containing both labels were analyzed with two-sided paired Wilcoxon signed-rank tests, followed by BH adjustment across six features separately within raw and z-scored analyses. Median paired differences and 95% confidence intervals were estimated from 10,000 bootstrap resamples of mouse pairs.

### 2.6 SGD classification and performance metric

Trace-based classification for the present analysis was reproduced in Weka 3.8.6 using weka.classifiers.functions. SGD (Hall et al., 2009; Frank et al., 2017). The first attribute was the nominal class label (itch or pain); neuron_id and mouse_id were excluded; and t1–t8438 were entered as numeric attributes. Each neuron therefore contributed one full-trace feature vector. The previously reported analysis used stratified random-neuron 10-fold cross-validation with seed 1, allowing neurons from the same mouse to occur in training and test folds. The Weka SGD options were -F 0 -L 0.01 -R 1.0E-4 -E 500 -C 0.001 -S 1, corresponding to hinge loss, learning rate 0.01, L2 regularization lambda 1.0E-4, 500 epochs, and classifier seed 1. The command retained -C 0.001, but this epsilon option is inactive under hinge loss. The normalized condition used Weka’s default numeric-attribute normalization; the non-normalized condition included -N.

Classification performance was summarized as mean class recall, defined as the average of itch recall and pain recall: Mean class recall = 1/2 × [(itch correctly classified/all actual itch) + (pain correctly classified/all actual pain)]. This metric is mathematically equivalent to balanced accuracy, but the term mean class recall is used throughout to make the calculation explicit. Gray error bars in performance plots indicate propagated 95% confidence intervals based on class-wise recall estimates. For each class-wise recall *r*, the standard error was estimated as sqrt[*r*(1 − *r*)/*n*], and the standard error of the mean class recall was propagated across the two classes.

### 2.7 Mouse-constrained shuffled-label control

Mouse-constrained shuffled-label analysis was used as a boundary-condition control for mouse-related structure. Labels were shuffled within mice containing both itch- and pain-associated responsive neurons. This procedure preserved mouse identity while disrupting the within-mouse association between response profile and condition label. Mice containing only one label were uninformative for within-mouse label exchange. The analysis therefore served as a mouse-constrained control rather than a complete permutation null distribution or complete removal of mouse-level dependence.

A repeated permutation sensitivity analysis restricted to the 14 mixed-label mice independently permuted labels within each mouse 1,000 times. A class-weighted logistic model using all six standardized event features was evaluated in seven mouse-exclusive folds, and the empirical upper-tail *p* value was calculated as (1 + number of null values at least as large as observed)/(1 + 1,000).

### 2.8 Mouse-level classifier validation and class-balance sensitivity

Cross-mouse transfer was evaluated using mouse-exclusive folds and a leave-one-mouse-out (LOMO) analysis restricted to the 14 mice containing both labels. In each LOMO fold, all neurons from one mouse formed the test set and the remaining 13 mixed-label mice formed the training set. Mean class recall was calculated for each held-out mouse and averaged with equal mouse weight; 95% intervals were estimated from 10,000 bootstrap resamples of mice.

Class-balance sensitivity was evaluated within each LOMO training fold by downsampling the majority label to the minority-label count while retaining the observed test-mouse composition. Five deterministic sampling repetitions were analyzed for each raw or *z*-scored representation and each Weka normalization setting. Training rows were randomized with recorded seeds before ARFF export, and external train/test Weka SGD runs used -force-batch-training to preserve the specified 500-epoch procedure.

### 2.9 Complementary classifiers and UMAP quantification

A nonlinear full-trace sensitivity analysis used Weka RandomForest with all 8,438 trace attributes, 100 trees, *K* = 0, minimum leaf size 1, and seed 1 under natural and balanced 10-fold mouse-exclusive training (Breiman, 2001). In Weka 3.8.6, *K* = 0 sets the number of randomly sampled predictors per split to floor(log2[*p*] + 1), where p is the number of predictors. Seven prespecified event-feature sets were evaluated under the same mouse-exclusive fold assignment: peak rate, peak interval, both timing features, both rise features, both decay features, all four kinetic features, and all six features. Linear feature models used batch Weka SGD with the settings specified above. An RBF-kernel Weka SMO classifier provided a nonlinear all-six-feature analysis with complexity parameter *C* = 1.0, filter type 0 (normalization), and gamma set to the inverse of the six-feature count (1/6). UMAP embeddings were generated separately from raw and neuron-wise z-scored full traces using Euclidean distance, 15 neighbors, minimum distance 0.1, and seed 1 (McInnes et al., 2018). Global and within-mouse label silhouette coefficients quantified separation in the displayed coordinates (Rousseeuw, 1987). A separate local-organization test projected each representation onto 50 principal components, selected 15 nearest neighbors within the same mouse, and compared the equal-weight mouse mean of excess same-label neighbors with 1,000 within-mouse label permutations.

### 2.10 Statistical and interpretive level

Neuron-level rank tests and random-neuron cross-validation describe differences and discrimination among recorded neurons. Paired mouse summaries evaluate within-mouse feature consistency, mouse-exclusive validation estimates transfer to an animal absent from training, and within-mouse permutation tests local label organization while holding mouse membership constant. Exact seeds, fold assignments, Weka commands and logs, prediction-level outputs, derived tables, and software-environment records are included in the reproducibility package.

## 3 Results

### 3.1 The ACC neurons show itch/pain-associated differences in calcium response features

In a previous study, trace-based SGD classification separated ACC neuron response profiles associated with itch- and pain-evoking conditions and a mean firing-frequency difference was reported (Ko et al., 2025). We therefore used the same denoised neuron-level calcium trace dataset to identify additional interpretable signal features that accompanied this separation (Figure 1A). Rather than treating the classifier as the central result, we analyzed peak-aligned transient morphology and event-level features in raw denoised and neuron-wise z-scored traces (Figures 1B–I). At the neuron level, itch- and pain-associated calcium response profiles differed across multiple response properties. The group-average raw signal and representative detected calcium peaks are shown in Figure 1B, whereas baseline-centered and peak-normalized average transients summarize group-level event morphology in Figure 1C. The primary detector identified 47,966 calcium events with identical event counts and peak indices in raw and z-scored traces. Figure 1B shows short windows from one Itch-labeled and one Pain-labeled neuron with the denoised input, smoothed trace, neuron-specific threshold, and accepted calcium peaks; Supplementary Figure S1 provides six additional examples. Peak rate and peak interval are displayed once using raw values because the corresponding *z*-scored values were identical for every paired neuron with a finite estimate. Feature distributions showed group differences in peak rate, peak interval, rise AUC, decay AUC, rise slope, and decay slope (Figures 1D–I). In raw denoised traces, rank-biserial effect sizes were modest but directionally informative: itch-associated neurons showed higher peak rate (Figure 1D; *r*_rb = 0.19), whereas pain-associated neurons showed longer peak intervals (Figure 1E; *r*_rb = −0.143), larger rise AUC (Figure 1F; *r*_rb = −0.060), larger decay AUC (Figure 1G; *r*_rb = −0.094), and less negative decay slope values (Figure 1I; *r*_rb = −0.120). Rise slope showed minimal separation in raw traces (Figure 1H; left, *r*_rb = 0.017; BH-adjusted *p* = 0.423), whereas *z*-scored traces showed a small difference (Figure 1H; right, *r*_rb = 0.061; BH-adjusted *p* = 0.004). Overall, itch-associated neurons exhibited higher event frequency, whereas pain-associated neurons showed longer inter-event intervals, larger rise and decay AUC values, and less negative raw decay slopes; rise-slope differences depended on the signal representation. Most feature differences persisted after neuron-wise z-scoring, arguing against a simple fluorescence-scale explanation. These feature differences provide interpretable correlates of the previously reported full-trace separation.

### 3.2 Neuron-level SGD classification separates itch- and pain-associated response profiles

We next tested whether complete neuron-level traces retained enough information to separate itch- and pain-associated response profiles. Ko et al. (2025) reported SGD separation of the two recording labels and a mean firing-frequency difference; the present analysis evaluated classification across raw, *z*-scored, normalized, and mouse-constrained conditions. Under the original labels, Weka SGD applied to raw denoised traces yielded a mean class recall of 0.746 with default normalization and 0.727 with normalization disabled. These performance estimates were obtained by 10-fold cross-validation rather than by evaluating the classifier on the same data used for fitting. *Z*-scored traces also supported classification (Figure 2A). For Weka SGD with default normalization, mean class recall was 0.746 for raw denoised traces and 0.742 for *z*-scored traces. For Weka SGD with normalization disabled, mean class recall was 0.727 for raw denoised traces and 0.742 for *z*-scored traces. Because neuron-wise z-scoring removes each neuron’s temporal mean and variance scale, preserved classification after *z*-scoring indicates that the separation contains information beyond simple amplitude scale. This transformation does not identify which temporal features the classifier used. The event-frequency, event-spacing, and transient-morphology analyses provide interpretable correlates of that separation. We next used mouse-constrained shuffled-label analysis to evaluate the contribution of within-mouse label-associated structure while preserving mouse identity (Figure 2B). Labels were shuffled within mice containing both labels, disrupting the within-mouse association between response profile and condition label. For raw denoised traces, Weka SGD with default normalization yielded a mean class recall of 0.539, and Weka SGD with normalization disabled yielded 0.544. For *z*-scored traces, Weka SGD with default normalization yielded 0.561, and Weka SGD with normalization disabled yielded 0.546. These lower values indicate that the original within-mouse label assignment contributes to classifier performance. This control addresses a simple mouse-identity explanation while retaining mouse-level dependence. UMAP of full traces was then used as a descriptive visualization of neuron-level response-profile organization (Figures 2C,D). The label-colored and mouse-separated views showed that the embedding was not visually attributable to one mouse alone, while formal interpretation remained based on the feature and classification analyses.

**Figure 2:**
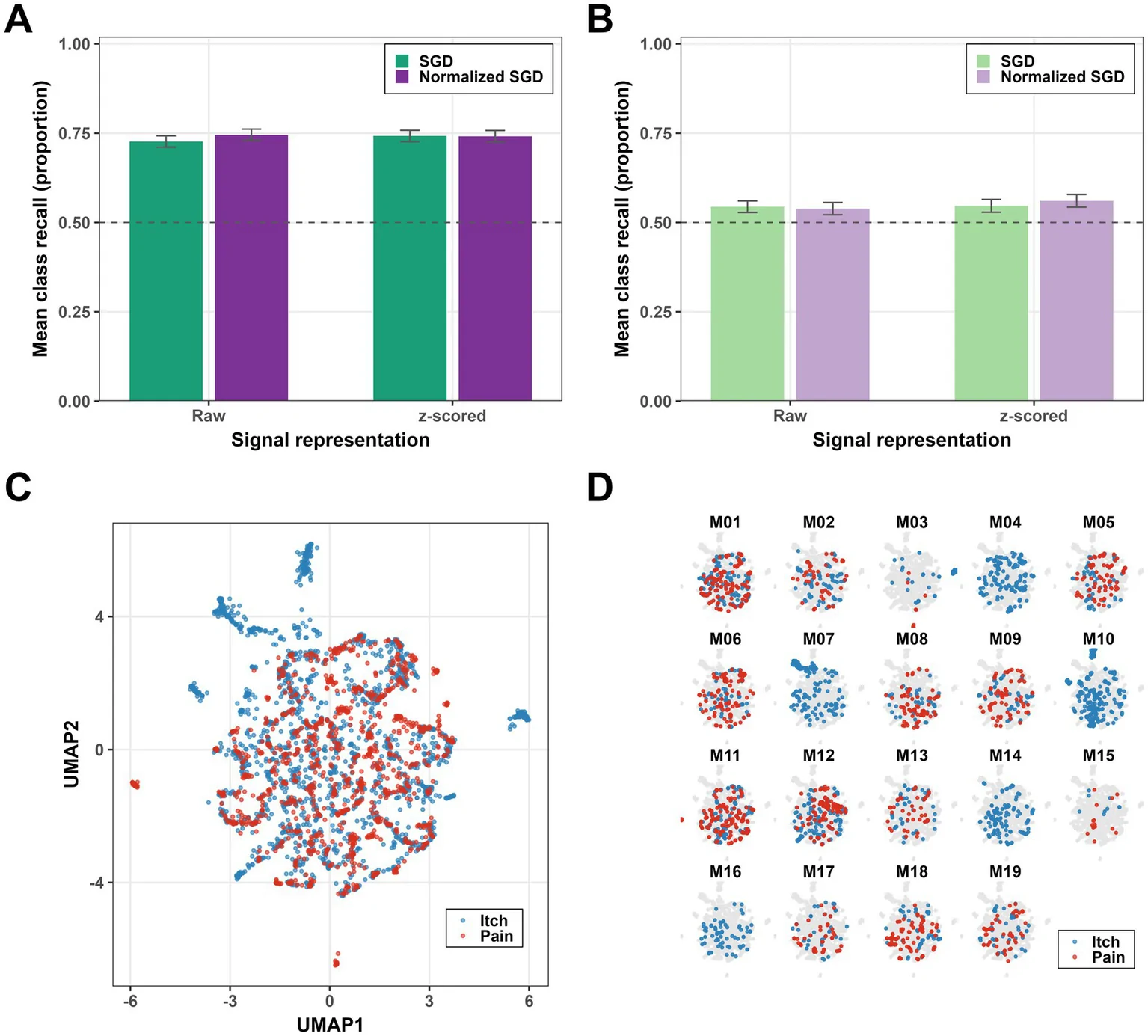
*Z*-scoring, mouse-constrained shuffled labels, and full-trace UMAP evaluate amplitude-scale and mouse-related explanations. **(A)** Mean class recall of Weka SGD classification using the original itch- and pain-associated labels in raw denoised and neuron-wise *z*-scored traces. SGD indicates Weka SGD with normalization disabled, and Normalized SGD indicates Weka SGD with default numeric-attribute normalization. Gray error bars indicate descriptive neuron-level propagated 95% confidence intervals based on class-wise recall estimates. **(B)** Mean class recall after the mouse-constrained shuffled-label control. Labels were shuffled within mice containing both itch- and pain-associated responsive neurons, preserving mouse membership while disrupting within-mouse label assignment. **(C)** UMAP visualization of neuron-wise *z*-scored full-trace response profiles colored by label. **(D)** The same embedding displayed separately by mouse, with all neurons in gray and neurons from each displayed mouse highlighted by label. Blue indicates itch and red indicates pain. Source M16 was absent from the supplied trace matrix before the present reanalysis; panel **(D)** retains the original display convention by labeling source mice M17–M20 as M16–M19. This relabeling applies only to the figure display. UMAP is descriptive; statistical inference is based on the feature and classifier-control analyses.

### 3.3 Mouse-level aggregation and class-balance sensitivity

Across the 14 mice containing both labels, the minimum BH-adjusted *p* values were 0.502 for raw traces and 0.270 for *z*-scored traces (Supplementary Figure S2; Supplementary Table S1). Mixed-label LOMO mean class recall was 0.469–0.493 across the four SGD conditions, with every 95% mouse-bootstrap interval spanning 0.5 (Supplementary Figure S3). Training-fold balancing produced five-repeat means of 0.477–0.490 and did not improve transfer to held-out mice. The neuron-level feature shifts and random-neuron classifier separation therefore did not establish a consistent mouse-level median shift or a classifier boundary that generalized across animals.

### 3.4 Complementary classifier analyses

Full-trace RandomForest mean class recall ranged from 0.496 to 0.538 under natural or balanced mouse-exclusive training, with all mouse-bootstrap intervals spanning 0.5 (Supplementary Figure S5). Balanced classifiers based on prespecified event-feature sets produced mean class recall values primarily between 0.505 and 0.579 (Supplementary Figure S6). The all-six-feature RBF-SMO model reached 0.566 for raw and 0.565 for *z*-scored features, with both intervals spanning 0.5. Repeated within-mouse label permutation of the all-six-feature logistic model yielded empirical *p* = 0.732 for raw and *p* = 0.526 for *z*-scored features (Supplementary Figure S7).

### 3.5 Quantified UMAP and within-mouse organization

Global label silhouette coefficients were 0.0026 for raw and 0.0049 for *z*-scored UMAP embeddings, with median within-mouse coefficients of 0.0101 and 0.0554 (Supplementary Figure S8). The two-dimensional display therefore showed weak label separation. In the prespecified 50-principal-component neighborhood analysis, the equal-weight excess same-label neighbor fraction was 0.042 for raw traces and 0.144 for *z*-scored traces. Positive excess occurred in 12 of 14 mixed-label mice for raw traces and all 14 for *z*-scored traces, with empirical upper-tail *p* = 0.001 for both representations after 1,000 within-mouse label permutations (Supplementary Figure S4). This analysis detected local label-associated organization while holding mouse membership constant.

## 4 Discussion

This study identifies small calcium-response profile differences among analyzed itch-associated and pain-associated ACC neurons. Itch-associated neurons showed more frequent peaks, whereas pain-associated neurons showed longer peak intervals, larger rise and decay AUC values, and less negative raw decay slopes. Rise-slope differences depended on the signal representation. These neuron-level differences appeared in raw denoised and neuron-wise *z*-scored traces (Figures 1B–I) and provide interpretable correlates of the previously reported full-trace classification.

Neuron-wise *z*-scoring subtracts each neuron’s temporal mean and scales the trace by its temporal standard deviation, reducing the influence of baseline offset and overall fluorescence scale. SGD classification remained effective after z-scoring (Figure 2A), demonstrating that discrimination persisted after removal of per-neuron mean and variance scale. This result establishes information beyond simple amplitude scale but does not assign the classifier decision to any single feature. The mouse-constrained shuffled-label control preserved mouse identity while disrupting the match between calcium trace and stimulus condition within mixed-label mice. The reduction in classification performance after this shuffle (Figure 2B) supports a contribution from within-mouse label-associated response structure.

The mouse-level analyses define the boundary of this neuron-level result. Paired mouse summaries did not detect a consistent median feature shift, and classifiers trained on other mice showed limited performance in a held-out animal even after training-class balancing. Conversely, the within-mouse neighborhood permutation detected local condition-associated organization after mouse membership was held constant. The results therefore support local label-associated organization within mice while limiting claims of cross-animal classifier transfer.

Pathway-specific mediodorsal thalamus-to-ACC activity has been linked to pain-related aversion in mouse neuropathic pain models (Meda et al., 2019), and Ko et al. (2025) reported segregated mediodorsal inputs to pain- and itch-associated ACC populations in the source study. These circuit findings provide biological context for condition-associated ACC response diversity. The present calcium-trace reanalysis contains neither circuit perturbation nor simultaneous electrophysiology, so it does not assign the measured feature differences to a specific pathway or biophysical mechanism.

Neural population geometry and shared dynamics can carry information beyond isolated single-cell summaries (Churchland et al., 2012; Cunningham and Yu, 2014; Vyas et al., 2020; Panzeri et al., 2022). The present measurements remain calcium-signal features. Peak interval, rise-to-peak AUC, decay AUC, and slope can reflect spike timing, burst probability, synaptic input, indicator kinetics, cellular calcium handling, or combinations of these processes (Grienberger and Konnerth, 2012). At 15 frames/s, the 66.7-ms sampling interval, five-frame smoothing, and 200-ms minimum event duration constrain interpretation to calcium-transient dynamics at subsecond-to-second scales rather than fine action-potential timing.

Model-based deconvolution methods such as OASIS infer latent neural activity through sparse non-negative deconvolution, whereas supervised approaches such as CASCADE infer spike rates using ground-truth electrophysiology and calcium datasets (Theis et al., 2016; Friedrich et al., 2017; Rupprecht et al., 2021). The current recordings lack simultaneous electrophysiology from the imaged neurons. Deconvolution would therefore constitute an additional model-dependent hypothesis test rather than independent validation of spike timing or firing-rate magnitude. The conclusions remain at the calcium-signal level.

Ko et al. (2025) established full-trace classification, a mean firing-frequency difference, and circuit-level segregation of itch- and pain-associated ACC populations. The present study extends that foundation by resolving peak interval and rise/decay morphology and by evaluating raw and z-scored representations with paired mouse summaries, mouse-exclusive validation, complementary classifiers, and within-mouse permutation. The resulting evidence supports small neuron-level feature differences and local within-mouse organization, with limited consistency of mouse-level medians and cross-animal classifier transfer.

## Data Availability

The raw data supporting the conclusions of this article will be made available by the authors, without undue reservation.
