# Supplementary material for: Distinct calcium responses distinguish itch- and pain-associated neurons in the anterior cingulate cortex
Source: Front Behav Neurosci. 2026 Oct 27;20:1948042. doi: 10.3389/fnbeh.2026.1948042 (PMC13748434; doi:10.3389/fnbeh.2026.1948042)
Supplement: Supplementary file 1 [file Supplementary_file_1.docx]

Distinct calcium responses distinguish itch- and pain-associated neurons in the anterior cingulate cortex

**Chiwoo Lee^1^, Hyoung-Gon Ko^1^***

^1^Department of Oral Anatomy and Neurobiology, Kyung Hee University College of Dentistry, Seoul, Republic of Korea.

*** Correspondence:**
Hyoung-Gon Ko
hgko@khu.ac.kr

Keywords: anterior cingulate cortex, itch, pain, calcium imaging, calcium transient

**Supplementary Figure S1. Calcium-event detection quality control.** Six single-neuron raw denoised traces are shown over 180 s from the beginning of the post-injection recording. Colored lines show the smoothed traces, faint gray lines show the denoised inputs, dashed lines indicate neuron-specific mean-plus-two-standard-deviation thresholds, and open circles mark accepted calcium peaks after the minimum-duration and merge-gap criteria. Blue traces are Itch-labeled and red traces are Pain-labeled.

**Supplementary Figure S2. Paired mouse-level feature summaries.** Each point is the median across neurons within one mouse-label combination, and gray lines connect Itch and Pain values from the same mixed-label mouse. Raw and z-scored values are shown separately for all six prespecified features. All 12 BH-adjusted paired comparisons exceeded 0.05.

**Supplementary Figure S3. Full-trace classifier performance across validation units.** (A) Raw denoised and (B) neuron-wise z-scored summaries. Random-neuron 10-fold values quantify discrimination among neurons in the assembled dataset. Mixed-mouse leave-one-mouse-out (LOMO) values are equal-weight means across the 14 mice containing both labels; error bars are 95% mouse-bootstrap intervals. Balanced mixed-mouse LOMO downsampled the majority class in training folds only; error bars show the range across five deterministic sampling repetitions. (C) Raw and (D) z-scored mouse-specific mean class recall under natural mixed-mouse LOMO. Purple indicates Weka SGD with default numeric-attribute normalization, and teal indicates Weka SGD with normalization disabled. The dashed line marks mean class recall of 0.5. Source mouse IDs were retained in analysis; displayed IDs follow the original sequential convention after source M16 was absent from the supplied trace matrix.

**Supplementary Figure S4. Mouse-equal local label organization in high-dimensional trace space.** (A) Raw and (B) neuron-wise z-scored mouse-specific excess same-label neighbor fractions. For every neuron, the 15 nearest neighbors were selected from the same mouse in a 50-principal-component space. Excess is the observed same-label fraction minus the analytical expectation from that mouse's label counts. Dotted horizontal lines mark the equal-weight mean across 14 mixed-label mice. (C) Raw and (D) z-scored null distributions from 1,000 independent within-mouse label permutations. Solid colored lines mark the observed equal-weight mean, and dashed gray lines mark the 2.5th and 97.5th percentiles of the null distribution. Empirical upper-tail P=0.001 for both representations. Source mouse IDs were retained in analysis; displayed IDs follow the original sequential convention after source M16 was absent from the supplied trace matrix.

**Supplementary Figure S5. Nonlinear full-trace classification sensitivity analysis.** Weka RandomForest used all 8,438 trace attributes and 100 trees under natural and training-fold-balanced mouse-exclusive validation. Error bars show 95% mouse-cluster bootstrap intervals, and the dashed line marks mean class recall of 0.5.

**Supplementary Figure S6. Mouse-exclusive classification using prespecified calcium-event features.** Points show mean class recall and 95% mouse-cluster bootstrap intervals under balanced mouse-exclusive validation. Teal points show linear Weka SGD feature sets; orange points show the RBF-kernel Weka SMO sensitivity analysis using all six features. The dashed line marks 0.5.

**Supplementary Figure S7. Repeated within-mouse label-permutation analysis.** Histograms show 1,000 mouse-constrained label permutations from an all-six-feature class-weighted logistic model evaluated in seven mouse-exclusive folds. Vertical lines indicate observed mean class recall. Empirical upper-tail P values were 0.732 for raw and 0.526 for z-scored features.

**Supplementary Figure S8A. Raw-trace UMAP displayed by mouse.** Each panel highlights neurons from one displayed mouse in the common raw-trace UMAP coordinates while retaining all other neurons in gray. Blue indicates Itch and red indicates Pain. Source mouse IDs were retained in analysis; source M17–M20 are displayed as M16–M19 because source M16 was absent from the supplied trace matrix. UMAP provides a descriptive display; silhouette diagnostics and the 50-principal-component neighborhood permutation quantify label organization.

**Supplementary Figure S8B. Neuron-wise z-scored UMAP displayed by mouse.** Each panel highlights neurons from one displayed mouse in the common z-scored UMAP coordinates while retaining all other neurons in gray. Blue indicates Itch and red indicates Pain. Source mouse IDs were retained in analysis; source M17–M20 are displayed as M16–M19 because source M16 was absent from the supplied trace matrix.

Supplementary Figure S8C. Mouse-specific label silhouettes in the two-dimensional UMAP displays. Raw and z-scored values are connected within each of the 14 mixed-label mice; the dashed line marks zero. Values near zero indicate limited label separation in the displayed coordinates. Green, raw; purple, z-scored. Source IDs were retained in analysis; source M17-M20 are displayed as M16-M19 after exclusion of source M16.

**Supplementary Table S1. Paired mouse-level feature statistics**

Values are medians across the 14 mice containing both labels. Difference is Itch minus Pain. Confidence intervals are percentile intervals from 10,000 mouse-pair bootstrap resamples. BH P values were adjusted across six features separately within each signal representation.

**Raw**

| **Feature** | **Itch** | **Pain** | **Difference** | **95% CI** | **BH P** |
| --- | --- | --- | --- | --- | --- |
| Peak rate | 0.017 | 0.018 | -4.44e-04 | -0.003 to 0.004 | 0.950 |
| Peak interval | 32.542 | 27.883 | 4.667 | -3.033 to 8.833 | 0.502 |
| Rise AUC | 0.018 | 0.022 | 0.002 | -0.006 to 0.007 | 0.950 |
| Decay AUC | 0.031 | 0.032 | -2.77e-04 | -0.008 to 0.009 | 0.950 |
| Rise slope | 0.022 | 0.026 | -0.003 | -0.009 to 0.003 | 0.693 |
| Decay slope | -0.014 | -0.015 | 0.002 | -3.37e-04 to 0.003 | 0.502 |

**Z-scored**

| **Feature** | **Itch** | **Pain** | **Difference** | **95% CI** | **BH P** |
| --- | --- | --- | --- | --- | --- |
| Peak rate | 0.017 | 0.018 | -4.44e-04 | -0.003 to 0.004 | 0.814 |
| Peak interval | 32.542 | 27.883 | 4.667 | -3.033 to 8.833 | 0.335 |
| Rise AUC | 2.382 | 2.263 | 0.138 | -0.014 to 0.413 | 0.270 |
| Decay AUC | 3.579 | 3.130 | 0.244 | 0.016 to 0.688 | 0.270 |
| Rise slope | 2.651 | 2.652 | -0.031 | -0.299 to 0.158 | 0.814 |
| Decay slope | -1.491 | -1.628 | 0.017 | -0.081 to 0.113 | 0.814 |

**Supplementary Table S2. Trace-classifier validation summary**

Mean class recall is the arithmetic mean of Itch and Pain recall. Grouped intervals use 10,000 mouse-cluster bootstrap replicates. Balanced grouped values are means across five training-fold-only downsampling repetitions, and their across-seed variation is reported as descriptive subsampling sensitivity.

| **Validation** | **Signal** | **Weka normalization** | **Mean class recall** | **95% CI or summary** |
| --- | --- | --- | --- | --- |
| Random-neuron | Raw | Default | 0.746 | - |
| Random-neuron | Raw | Disabled | 0.727 | - |
| Random-neuron | Z-scored | Default | 0.742 | - |
| Random-neuron | Z-scored | Disabled | 0.742 | - |
| Mouse-exclusive | Raw | Default | 0.509 | 0.489-0.530 |
| Mouse-exclusive | Raw | Disabled | 0.513 | 0.486-0.539 |
| Mouse-exclusive | Z-scored | Default | 0.510 | 0.465-0.551 |
| Mouse-exclusive | Z-scored | Disabled | 0.502 | 0.474-0.528 |
| Mouse-exclusive balanced | Raw | Default | 0.497 | five-repeat mean |
| Mouse-exclusive balanced | Raw | Disabled | 0.514 | five-repeat mean |
| Mouse-exclusive balanced | Z-scored | Default | 0.503 | five-repeat mean |
| Mouse-exclusive balanced | Z-scored | Disabled | 0.507 | five-repeat mean |

**Supplementary Table S3. Mixed-label leave-one-mouse-out Weka SGD summary**

Natural-training values are equal-weight means across 14 held-out mixed-label mice with 95% intervals from 10,000 mouse bootstrap resamples. Balanced-training values are means and ranges across five deterministic training-fold downsampling repetitions. Each test mouse retained its observed neuron counts.

| **Signal** | **Weka normalization** | **Natural macro MCR** | **Mouse-bootstrap 95% CI** | **Median mouse MCR** | **Balanced mean MCR** | **Balanced repeat range** |
| --- | --- | --- | --- | --- | --- | --- |
| Raw | Default | 0.469 | 0.431-0.505 | 0.476 | 0.487 | 0.478-0.498 |
| Raw | Disabled | 0.470 | 0.434-0.508 | 0.458 | 0.477 | 0.470-0.485 |
| Z-scored | Default | 0.469 | 0.422-0.511 | 0.498 | 0.486 | 0.472-0.501 |
| Z-scored | Disabled | 0.493 | 0.454-0.530 | 0.506 | 0.490 | 0.482-0.497 |
